# Acceptability and Scalability of a Meditation App Among Adolescents With Type 1 Diabetes Mellitus

**DOI:** 10.7759/cureus.72700

**Published:** 2024-10-30

**Authors:** Ejura Y Salihu, Helen Omuya, Deborah T Joseph, Judith H Hassan, Asma Ali, Betty Chewning

**Affiliations:** 1 School of Pharmacy, Social and Administrative Sciences Division, University of Wisconsin-Madison, Madison, USA; 2 Department of Family Medicine and Community Health, University of Wisconsin-Madison, Madison, USA; 3 Department of Community Health, Hospital Sisters Health System, Springfield, USA; 4 Department of Health Sciences and Social Work, Western Illinois University, Macomb, USA; 5 School of Public Health, The University of Memphis, Memphis, USA

**Keywords:** adolescents, healthy minds program app, meditation, meditation app, mental health applications (mhapps), type 1 diabetes

## Abstract

Background

Adolescents with type 1 diabetes mellitus (T1DM) experience stress from general life stressors and diabetes-specific stressors. This stress manifests in a range of ways, such as mood swings, heightened frustration, strained familial relationships, and difficulties in T1DM self-management, which then leads to worse health outcomes. There is small to moderate evidence that frequent use of mental health applications (MHapps) improves mental and physical health outcomes. Meditation apps may help reduce some of the stress associated with living with T1DM. This study explores the acceptability and scalability of a self-guided, smartphone-based meditation app, the Healthy Minds Program (HMP) app, among adolescents with T1DM using the Unified Theory of Acceptance and Use of Technology.

Methods

Eight adolescents ages 15-19 were recruited from a pediatric clinic in a Midwestern state and introduced to the HMP app. After using the HMP app for one week, they were invited to participate in three successive focus group meetings. During the meetings, they shared their perspectives on the content, navigation, and acceptability of the HMP app and strategies to introduce and scale app utilization among adolescents with T1DM. Researchers conducted conventional content analysis using a hybrid coding approach. Data was managed and analyzed using NVivo 10 (Lumivero, Denver, Colorado, USA).

Findings

Participants believed that the HMP app has the potential to enhance their stress management, mood, and coping abilities when dealing with the challenges of T1DM management. They found the app enjoyable and easy to use but expressed concerns about time constraints as a potential barrier. To address this, they shared recommendations for facilitating app uptake and usage.

Conclusions

This study’s results provide an in-depth understanding of how positively this subset of adolescents with T1DM viewed the HMP app. The participants also offered valuable suggestions that can promote the adoption and sustained use of MHapps by adolescents living with T1DM.

## Introduction

In the last decade, there has been a rise in the number and use of mental health applications (MHapps) [1-3]. Most MHapps provide users with mental health information and/or an opportunity to monitor and manage their mental health [2,4]. Researchers attribute the surge in MHapp usage to its role in normalizing mental health while addressing some of the limitations of traditional mental health care [3,5]. Conventional approaches to mental healthcare are insufficient because of challenges such as limited availability of mental healthcare providers, low accessibility for people in lower socioeconomic classes, and low social acceptance in hard-to-reach populations [5,6]. MHapps are accessible, cost-effective, and offer privacy for effectively managing mental health with less stigma [1,3,4]. Studies have shown that MHapps are expanding the reach of mental health and wellbeing services, particularly for young people [7,8]. Despite the widespread interest in MHapps, many remain underutilized [8,9]. Studies of MHapps found that over half of MHapps had no regular users over one month [8].

Mental illness is one of the most common health challenges faced by young people between the ages of 10 and 24 [10], but adolescents living with chronic illnesses such as type 1 diabetes mellitus (T1DM) face markedly elevated rates of mental health illness compared to their peers [10-12]. Adolescents with T1DM experience short-term and long-term stress from general life stressors and diabetes-specific ones. This stress can manifest in a range of ways, such as mood swings, heightened frustration, strained familial relationships, and difficulties in T1DM self-management, which then lead to worse health outcomes [12,13]. Meditation-based MHapps may help reduce some of the stress associated with diabetes. Previous studies have shown their potential for improving mental and physical health outcomes [2,8,14-17].

Several studies show that MHapps receive high acceptability among adolescents and young adults due to their accessibility [7,8,15]. To maximize the benefits of MHapps, adolescents must consistently use these apps over an extended period [7,8,15]. Unfortunately, long-term MHapp use continues to be less than optimal. [8,9]. Adolescents living with T1DM are a vulnerable group in need of innovative, tailored mental health support [10-13]. Designing MHapps specifically for adolescents with T1DM is crucial, as these apps could provide much-needed relief from these challenges.

The Healthy Minds Program (HMP) app is a self-guided meditation MHapp designed by Healthy Minds Innovation, Inc. [18]. The app includes podcast-style lessons on the science of training the brain and meditation sessions intended to cultivate mental and emotional skills associated with emotional well-being (Awareness, Connection, Insight, and Purpose). Users can choose the format (active versus seated versions) and the duration of meditation sessions they prefer (session duration ranges from five minutes to 30 minutes). The app also has an assessment feature called the Healthy Minds Report. The report allows users to measure their current level of well-being and see how it changes over time. Studies of the app have yielded positive results in different populations in the USA, with a randomized controlled trial (RCT) of the HMP app with adults showing reduced psychological distress and improved outcomes related to well-being. This success has led to the recommendation of using the HMP app in other RCTs to improve the quality of life in individuals living with chronic illnesses [18].

Another study explored the impact of the HMP app on mental health and other well-being outcomes in medical students in a Midwestern state in the USA. The study results showed a significant reduction in psychological distress and a high rate of app usage among participants [19]. These results highlight the potential of the HMP app as an acceptable tool for reducing distress in young adults. Despite the success of these studies, to our knowledge, this study is the first to explore the acceptability of the HMP app among adolescents living with T1DM. This study aims to get preliminary data on adolescents’ engagement with the HMP app, the extent to which they believe their use of the app helps manage some of the stress associated with managing T1DM, and strategies for promoting app uptake and utilization among this population.

The Unified Theory of Acceptance and Use of Technology (UTAUT) is a framework used to examine individuals’ intentions to use technology and potential for subsequent use [20]. The theory has four major domains: Performance Expectancy, Effort Expectancy, Social Influence, and Enabling Conditions. The first three are direct predictors of user intention and behavior, while the fourth is a predictor of only user behavior. Several studies have shown that the impact of the four constructs on user intention and behavior is moderated by gender, age, experience, and voluntariness of use [20].

The UTAUT model has been widely used and validated for predicting technology adoption and usage decisions in various fields, including health research [20]. For this study, Performance Expectancy was operationalized based on the perceived usefulness of the HMP app’s perceived impact on diabetes distress, and general well-being. Effort Expectancy was operationalized as interest and enjoyment in using the app and perceived app functionality about managing T1DM. Social Influence was defined as external factors influencing app usage, such as peer support and family members’ experience with apps. Enabling Conditions was operationalized as personal facilitators and barriers to continued app use. Social Influence was merged with enabling conditions due to similarities in both themes.

The objective of this study was to gather preliminary data on adolescents’ engagement with the HMP app and their perceptions of how it aids in managing stress related to T1DM. Additionally, the study aimed to identify effective strategies for encouraging adolescents with T1DM to adopt and use the HMP app.

## Materials and methods

Study design

This qualitative study is part of a larger study [21] that explored adolescents’ perspectives on diabetes distress and the potential role of the HMP app to reduce distress and improve self-management in adolescents with T1DM. The sample was drawn from a cohort receiving diabetes care at one Midwestern health system who had agreed to be contacted for research. All participants identified as non-Hispanic Whites. In the first phase, adolescents participated in a semi-structured interview where they answered questions regarding their experiences living with T1DM, psychological barriers and facilitators of self-management, beliefs about meditation practice in general, and specific interest in using MHapps [21]. Regardless of the participant’s previous experience with meditation or lack of it, at the end of the interview, the researcher invited them to participate in the second phase of the study, which is presented in this paper.

Eight adolescents were asked to participate in this study. As with the larger study, participants were included if they were between 15 and 19 years old, diagnosed with T1DM for more than one year, willing to use the HMP app, and able to read and speak English fluently. The research team guided participants through the app installation process on their phones. They were encouraged to use the app daily for at least one week before the first focus group meeting. Each focus group meeting was held at least one week apart. Before each meeting, participants were asked to continue using the app and prepare ideas to share with the group.

Data collection

Five adolescents participated in the three successive focus group meetings after using the HMP app. During the first focus group, participants answered questions about how they used the app, the role they thought it might play in emotional well-being, and some potential challenges with using it. In the second meeting, participants shared their opinions on different potential settings for implementing the app, including suggestions for potential research collaborators. Finally, in the last meeting, the participants shared their feedback on the study design, including sample and recruitment strategies and the outcome measures for future HMP-based interventions.

A trained qualitative researcher (EYS) and an expert in qualitative research and focus group facilitation (BC) conducted three 75-minute focus group meetings with participants. A third researcher (AA) observed the meetings, taking notes of non-verbal cues and asking clarifying questions when needed [22]. The meetings occurred via a secure web-based meeting platform (WebEx, Cisco Systems, San Jose, California, USA) from August 2021 to September 2021.

The focus group approach was used for this study to encourage participants to develop a sense of belonging in the group and interact with one another’s ideas and opinions, which generated rich data while being time efficient [22]. The focus group guide (Appendix A) included open-ended questions allowing participants to share their in-depth perspectives on the different aspects of the HMP app (including navigation, user interface, and content) and recommendations for introducing and facilitating app use in different settings. They also shared their opinions on developing and implementing HMP-based interventions for this population. Meetings were audio-recorded and transcribed verbatim.

Data analysis

Researchers (EYS and HO) used deductive and inductive open-coding approaches to conduct a directed content analysis [23]. Both researchers coded the transcripts primarily with a categorization matrix that was derived from the UTAUT framework but allowed inductive codes to emerge from the data. The researchers read the transcripts line by line and independently coded the data, then met to discuss similarities and differences. Codes were consolidated into categories, and these categories were then organized into relevant themes to answer the research questions [23]. Data management and analysis were done using NVivo 10 (Lumivero, Denver, Colorado, USA). To ensure rigor, researchers used Lincoln and Guba’s criteria for establishing trustworthiness in qualitative research [24]. Thick descriptions of the study design, data collection, and analysis have been provided to aid the transferability of findings to similar cultural contexts. Member checking was also conducted by sharing the results with the participants to ensure the accuracy of quotes and interpretations [24].

This study was approved by the University of Wisconsin-Madison Institutional Review Board (approval number 2021-0668).

## Results

This section presents themes that emerged from the focus group discussions, with participants referenced using identifiers (such as P1). A summary of the themes and subthemes derived from the data is presented in Table 1.

**Table 1:** Themes and subthemes related to perspectives on acceptability and scalability of HMP app among adolescents with T1DM HMP, Healthy Minds Program; T1DM, type 1 diabetes mellitus

Themes	Subthemes
Performance expectancy of the HMP app	Supports management of diabetes-related stress in the early stages of diagnosis
	Prevents mood problems and improves the mental state
	Aids coping for dealing with overwhelming diabetes-related information and pressures
Effort expectancy of the HMP app	Easy navigation of the HMP app
	Variety of meditation sessions on the HMP app
	Presence of an ongoing well-being assessment feature
Enabling conditions of the HMP app	Facilitators of HMP app usage
	Potential challenges associated with utilizing the HMP app
Recommendations for designing and implementing the HMP app to study it for adolescents with T1D	Key elements to include in an HMP app-based study
	Strategies to introduce the app to the target population
	Establishing eligibility criteria for the target population with specific demographic characteristics

Focus group 1: role in emotional well-being

Adolescents’ opinions of the HMP app were grouped into the four UTAUT domains: Performance Expectancy, Effort Expectancy, Social Influence, and Enabling Conditions. Performance Expectancy included their beliefs about the impact of HMP app usage on diabetes distress, T1DM self-management, and general well-being. Effort Expectancy included their opinions on the ease of the app navigation and features, including the variety and length of meditation sessions built into the app. Social Influence focuses on social factors affecting app usage, such as family and peer influence. Enabling Conditions included facilitators (prior meditation experience and personal need for meditation), and barriers (such as competing demands for time). During analysis, it became evident that participants in this study believed social influences such as family and peers were an important influence facilitating their app uptake (e.g., parents who meditate frequently encouraged their children to also meditate, and children with parents or friends who meditate believe that they also need meditation). Given the close relationship between the Social Influence and Enabling Conditions themes, both themes were merged and labeled Enabling Conditions. Other themes that arose using inductive analysis included participants’ suggestions for strategies to increase app uptake, utilization, and evaluation among adolescents with T1DM.

Theme 1: Performance Expectancy of the HMP App

Participants anticipated outcomes from using the HMP app, including how the app could help them manage diabetes-related stress, prevent mood problems, improve their mental state, and aid in self-management of T1DM through improved coping.

Subtheme 1: Supports management of diabetes-related stress in the early stages of diagnosis: Adolescents described the challenges associated with effectively managing T1DM, particularly for individuals newly diagnosed with the disease. They believed the HMP app might be useful for dealing with the numerous stressors associated with managing T1DM and help them adapt to the lifestyle changes associated with being newly diagnosed with T1DM.

*I see how using the HMP app is helpful for people with diabetes because managing diabetes is stressful. I feel like it is more useful for people newly diagnosed with diabetes because they are stressed and figuring out that their life is now switched *- P5

Subtheme 2: Prevents mood problems and improves the mental state: Participants believed that the app has great potential to help them get into the habit of meditation and consequently improve their overall mental well-being. They stated that using the app frequently will help them steer away from negative mindsets that adversely impact their self-management and ability to cope with both diabetes-related and general life stressors.

*It is something I wanted to get into for a while because I feel like meditation does help the mind, and it helps keep people out of bad mindsets and out of bad spots… If I start my day off by doing meditation with this app, it puts me in a good mindset for the rest of the day* - P1

*I see how using the HMP app will help improve one’s mental state* - P3

Subtheme 3: Aids coping for dealing with overwhelming diabetes-related information and pressures: All the participants agreed that the HMP app would be a useful tool for adolescents dealing with the pressures and expectations of managing T1DM. The group unanimously agreed that adolescents with T1DM experience significant pressure from dealing with all the lifestyle changes and being overwhelmed by the sheer volume of information on T1DM and self-management tasks. Participants believe that the app will help them process their emotions and handle these expectations with more patience.

*I think it [the HMP app] would be useful for most teenagers with diabetes to utilize, as teenagers nowadays are, like, presented with a lot of information and a lot of pressures, especially with diabetes, and expecting us all to be able to handle that like gracefully without falling apart at the seams. I think it [the HMP app] would help management and help people to be able to clear their headspace and be able to process these feelings a little more* - P5

Theme 2: Effort Expectancy of the HMP App

The primary themes identified in the effort expectancy of the HMP app were navigation, variety, and assessment. Participants found the app user-friendly because it was easy to navigate. They enjoyed the variety of sessions and found the Healthy Minds Report to be beneficial.

Subtheme 1: Easy navigation of the HMP app: Participants mentioned that they found the app easy to navigate. They also stated that the app was stylish and appealing, which added to its ease of use. Overall, participants found the app user-friendly.

*I like the whole style and pace of the app. It is really easy to navigate* - P1

Subtheme 2: Variety of meditation sessions on the HMP app: Participants also observed the app’s versatility and appreciated the array of options and categories of meditation sessions, which made it engaging and exciting to use.

*The [HMP] app gives you lots of options, so it is not like listening to the same thing over and over. The app is not boring to use, and it keeps track of the days you used it* - P3

Subtheme 3: Presence of an ongoing well-being assessment feature: Participants also liked having the Healthy Minds Report (which contained pre- and post-assessment scores). They believed the report was beneficial, allowing them to assess and monitor their well-being over time.

*I would say that the assessment, in the beginning, is really helpful and makes it easier to track your progress as you go along* - P5

Theme 3: Enabling Conditions of the HMP App

Adolescents outlined several factors that might contribute to adopting the HMP app. These factors include previous meditation experience, social influence from peers, and personal need for meditation.

Subtheme 1: Facilitators of HMP app usage: Previous experience with meditation. Participants shared that having prior exposure to meditation, particularly being introduced to it by people they admire and listen to, such as teachers and parents, increased their likelihood of using the app.

*The way that most of us got introduced to things like meditation and yoga and stuff was our parents being like, ‘Hey, I used to do this; you should try it too”* - P3

*Our teacher used to teach yoga and meditation, and our school is super into meditation, so we dabble in meditation* - P5

Personal need for meditation. Participants recognized the impact of using the app to meet their personal need for meditation. In addition to its potential to manage diabetes-related stress, they believed that the app would be beneficial for helping calm them down when they feel overwhelmed with diabetes self-management tasks or when they have other daily life-related mental stress.

*This meditation was something I wanted to start doing because of mental health issues, and meditation I thought would help me settle down if I was ever in a bad state* - P1

*I would like to relieve some stress *- P9

Subtheme 2: Potential challenges associated with utilizing the HMP app: Participants had concerns with incorporating the app into their hectic schedules. Notably, time management was the biggest issue for most of the adolescents in this study.

Competing demands on time. Some participants noted their worry that their busy lifestyle might hinder their ability to find time for meditation using the HMP app.

*I would suggest the HMP app to other people to see if it helps them, but I feel like myself; I have a lot going on. Usually, I do not have a lot of free time in general, so I don’t know, but it is worth a shot, I guess* - P6

*I think one of the big things for me is not like which meditation I do but like having a specific time to do it [app] so that I remember* - P3

Focus groups 2 and 3: feedback on future studies

Theme 5: Recommendations for Designing and Implementing the HMP App to Study It for Adolescents With T1DM

The participants shared suggestions to increase app uptake, utilization, and evaluation, such as recruitment strategies, choosing an eligibility criterion for the target population, and key elements to include in a future HMP app study. They also offered recommendations on how to facilitate meditation habit formation in participants, study design, including whether or not to include parents in the study, and ways to assess health outcomes.

Subtheme 1: Key elements to include in an HMP app-based study: Incorporate medication adherence strategies to facilitate meditation habit formation. Participants emphasized the importance of integrating medication use habits into the HMP app to facilitate the formation of meditation habits. Suggestions included setting alarms or reminders that align with medication schedules to make it easier to remember both tasks.

*You could always put meditate slash meds when you set your alarm. That’s like trying to meditate at the same time of the day when you do your sugars. You know, “I will try to do my sugar monitoring when I want to use the app.” In a way, it’s your timestamp *- P1

Expand the definition of well-being in the HMP app evaluation. Participants discussed the need to expand the measurement of well-being when conducting research with adolescents with T1DM. The group associated wellbeing with characteristics such as getting good grades in school, being knowledgeable about their health and feelings towards it, adhering to self-care routines, and being resilient in the face of adversity. Physical activity and organizational skills were also mentioned as key indicators of well-being that they would like to have seen more of in the Healthy Minds Report and/or other survey instruments that measure well-being in this population.

*[When I think of well-being], I think of someone with good grades who is smart. So someone who has routines and sticks to them. They can handle themselves…like if something goes wrong, they’re not gonna break down. A balanced person* - P3

*It should have slightly different questions for each category of a person like “I’ve type 2 diabetes and I want to focus on this”…I’d also add [to the survey instrument] “I’m aware of when I have negative feelings toward this blood sugar or something like that”… or “Do you feel secure in finding an insurance or health insurance?... Are you scared? Questions like these *- P3

Include opportunities to connect with others and use the HMP app with others. Adolescents stated that social factors such as family and peer influences play a role in their acceptability of the app. They believed that using the app in a group setting, potentially with friends, could provide motivation and encouragement. They suggested that the app could be introduced to adolescents in a group setting to encourage everyone to meditate together instead of introducing it to just individuals. They opined that having everyone in the room at the same time would make them more inclined to pay attention to the app and encourage them to use it on their own later. However, they warned that some individuals might prefer meditating alone, so it might be beneficial to offer both options to promote inclusivity. Generally, participants believed that social influence would enhance their likelihood of adopting the HMP app in their daily lives.

*I think you could be more motivated if we did it [used the HMP app to meditate] in a group. But I know that some people would rather do it by themselves. So, if you get to choose how you want to meditate, I think that would help* - P2

Consider preferences for parental inclusion in the study. A significant consideration emerged regarding the inclusion of parents in the HMP study. Participants recognized that individual preferences varied as some expressed reservations about parental involvement. One participant suggested that parental involvement may not be beneficial for some people. Specifically, concerns were raised about strict parental beliefs and the desire for privacy.

*If you have strict parents who do not believe in some things, then you don’t want your parents to find out about it…it would not be a good idea to have your parents involved in the meditation app* - P1

*I think that would be kind of a personal preference for some people. For me, I do not think my parents would meditate themselves, and I do not know how helpful it would be to me to have them also use the app or just remind me or something. But then there could also be people who do not want their parents to be involved in it. Just have it be their own thing *- P6

Subtheme 2: Strategies to introduce the app to the target population: Recruit from locations frequented by adolescents with T1DM. Participants shared strategies for introducing the HMP app to the target population, including diabetes camps, clinics, and schools, and advertising it on social media platforms. Participants proposed that healthcare providers, such as nurse practitioners, introduce the app during clinic visits, making it part of routine diabetes care. Participants also recommended introducing the app during diabetes camps, where counselors and nurses could promote its use.

*If my health care provider introduced me to this, I would not be opposed to using it… it would be interesting to see them try and introduce this. And then, like, during a checkup, [say] “I see you have used the app a little bit; is it a good fit for your treatment?” *- P1

*I went to the Wisconsin Lions Camp, and they have two or three weeks dedicated to diabetes. All the counselors had diabetes, and the nurses there were Children’s Hospital-trained nurses. It might be a good idea to introduce the HMP app there* - P4

Social media platforms like Instagram and Snapchat were suggested as effective channels for promoting the app, given their potential to reach a younger audience.

*I would stick it [ad for app] on social media like Instagram or Snapchat. This is where you’d find more people who are more willing to utilize it *- P5

Determine readiness for intervention before introducing the HMP app to adolescents with T1DM. Participants provided insights into how to evaluate readiness for the intervention. They suggested that future studies include survey questions that assess self-efficacy and readiness for intervention. Specifically, the question proposed by P3 was, “How much am I willing to let other people help me with my diabetes?” The participants added that by asking this question, researchers can predict who will benefit most from the intervention. This suggestion was based on their belief that adolescents who rate themselves as not being willing to allow others to help them with managing T1DM are unlikely to enroll in or complete interventions designed to help them manage T1DM.

Subtheme 3: Establishing eligibility criteria for the target population with the specific demographic characteristics: The study participants suggested two approaches for developing eligibility criteria for the target population:

Include all youths in the intervention. Participants argued that meditation is beneficial for everyone, not just people with diabetes. They advocated for the broader inclusion of participants.

*Meditation is not just something for people with diabetes. It is something for everyone* - P1

*I think that obviously, meditation can be utilized in normal people’s lives daily to have good health, as well as people with diabetes. So, introducing the app to a wider range of participants would not be a bad idea* - P3

Focus on newly diagnosed people with diabetes. Some participants suggested that the HMP app might be more useful for newly diagnosed individuals with diabetes who are navigating significant life changes and experiencing stress.

*I feel like it is almost more useful for new diabetics because they have a lot more going on… they feel stressed and are figuring out that their life is switched. I think that this app is more helpful geared toward newer diabetics or insecure diabetics* - P5

In addition to offering insights to help frame future studies, these findings provide valuable insights for introducing the HMP app to adolescents living with T1DM with the goal of reducing diabetes distress and improving well-being and self-care.

## Discussion

This study is unique in that it has explored the perspectives of adolescents with T1DM on their engagement with the HMP app, the extent to which they believe their use of the app is helpful, and strategies for promoting app uptake and utilization among this population. They felt the HMP app has significant potential to help their mental health. More research is needed on this question, particularly in a time of scarce healthcare resources to assist with mental health issues.

Perceived benefits

Participants believe the HMP app is beneficial in reducing diabetes distress, improving their overall well-being, and enhancing their ability to cope with their T1DM diagnosis. These findings support previous research that examined the acceptability of meditation apps for improving psychological well-being. Some studies, such as the meta-analysis by Gál et al. (2021), found that participants frequently reported significant improvement in anxiety, depression, and stress levels after using meditation apps [25,26]. Participants’ reports about the usefulness of the HMP app for improving mental health align with the results of previous studies that examined the impact of the app on the general population [18,19].

Perceived usability and acceptability

Adolescents’ perceptions of the app’s usability were centered around three key aspects: ease of use, variety of meditation sessions, and additional features that made the app engaging. They also appreciated the variety of podcast-style lessons and active/seated meditations in the app. They believed that this variety made the app engaging and enjoyable. Participants found the HMP app user-friendly due to its intuitive design and layout. Most participants mentioned that their favorite element of the app was that it was self-paced, and each module (Awareness, Connection, Insight, and Purpose) was grouped nicely like a curriculum so that they could move quickly from one lesson and meditation practice to the next. They found this arrangement easy to navigate. Participants particularly enjoyed using the well-being assessment feature, as it provided baseline information to track their progress and well-being over time in a structured way. Overall, participants reported high acceptability of the HMP app based on the three metrics they chose: ease of use, variety of sessions, and additional features for assessment.

Settings and support for use

Previous studies like this one showed that parental and peer support strongly influence well-being and treatment adherence among adolescents with chronic conditions [27-29]. In this study, participants noted the merits of using the app with peers in a group setting, such as school or camp. Parent support was also viewed positively. Participants shared conflicting views on involving parents in the intervention. Participants’ views on peer support are similar to another study that found that peer support promotes better outcomes for young people [29]. Future research should explore how peer support can influence app uptake and the sustainability of its use in this population.

Time challenges for use

Despite the reported high acceptability, participants noted several challenges preventing consistent use of the app. Competing demands on time was the critical barrier. Participants suggested strategies to overcome this challenge, such as deliberately scheduling specific times throughout the day and week to use the app for guided meditation. However, participants expressed having schedules that were already full of school and work obligations, which might hinder their ability to develop the habit of meditation. Previous studies also found this barrier to be the primary hindrance to using MHapps [2].

Further research is needed to assess strategies to help adolescents identify or create a few regular opportunities to meditate (e.g., bedtime, after school in a car before driving home, first thing in the morning). The Habituation-Intention Framework may offer a helpful model in that it has helped embed tai chi practice into a person’s daily activities [30]. To enhance app utilization, participants suggested integrating the existing reminders to perform self-management tasks into the HMP app and to facilitate the formation of meditation habits.

Enhancing uptake and new users

Participants offered other suggestions to enhance app uptake, utilization, and evaluation. To improve the app uptake, participants suggested ways to introduce the app to the target population and introduce it in various settings, including diabetes camps, clinics, schools, and social media platforms. They emphasized the role of healthcare providers in promoting the app during clinic visits as part of routine diabetes care. Furthermore, participants debated whether the app should have a broader audience or focus specifically on people newly diagnosed with diabetes, acknowledging the varying needs and circumstances of individuals within this population. One possibility would be to introduce an app in the hospital after diagnosis. This offers the possibility of personal follow-up as a person tries it out, with potential coaching on creating a practice plan. Future studies can also explore integrating social support features in the app based on user preferences. Expanding the definition of wellbeing in the app’s evaluation was also proposed. Participants provided suggestions for creating evaluation measures of well-being to assess the impact of the HMP app among adolescents with T1DM, including academic success, health knowledge, self-care adherence, and resilience in the face of adversity.

Limitations

This study had some limitations, such as a small sample size and the fact that all participants received care from one Midwest healthcare system. While this allowed us to get valuable data, we did not include adolescents who were experiencing severe diabetes-related mental health issues or those not already receiving diabetes care. Additionally, there may be self-selection bias, as participants who chose to enroll in this study may have already held positive attitudes toward meditation. Future work could consider studying broader and larger samples that include adolescents diagnosed with diabetes distress and other mental health illnesses. Despite these limitations, this study provides valuable insights from a subset of adolescents with T1DM regarding their experiences and preferences for a meditation app designed to help them manage the stress associated with living with T1DM. The findings from this study can be used to refine the HMP app to meet the needs of this population. The findings can also lead to promising directions for developing and implementing future MHapps studies on managing diabetes distress and improving emotional well-being in adolescents with T1DM.

## Conclusions

The results of this study provide an in-depth understanding of how positively this subset of adolescents with T1DM viewed the HMP app. They also offer valuable guidance for future interventions to reduce diabetes-related distress and improve overall well-being and self-care among this population. These findings can be useful in future studies that aim to employ tailored strategies to promote the adoption and sustained use of MHapps by adolescents living with T1DM.

## Appendices

Appendix A

Focus Group 1 Guide

Introduction

Hello and welcome. Thank you for participating in this focus group today.  My name is ____. I am a student at the _________. I will be leading our discussion. With me, today is ______. She will help me lead our discussion session today. We would like to thank you for your time and for sharing your thoughts during your interviews. We got a lot of helpful insights that would help us. This meeting will last for 90 minutes, and I will be recording our discussion so I don't miss any of your important comments. All your comments will be strictly confidential, and if we write any reports or publications about this group, we will not use your names.

**Share a slide of the group norms and expectations

"We value everyone's voice, so please share your thoughts and opinions freely. Please raise your hand if you want to speak so that everyone can have a chance to speak. This is a safe space, so feel free to share your opinions and allow others to speak freely too. Please do not disclose what we say in this group to anyone after this meeting."

Icebreaker question: Please state your first name, "one thing" that you are really good at, and use an emoji from the bottom of the screen to describe how you’re feeling right now

Now that we have all introduced ourselves let's dive in. Today we'll be talking about a mindfulness meditation app that I sent to you when we spoke.

We would like to hear your thoughts on the app and how it can be implemented in T1D management to improve emotional well-being and adherence.

Before we proceed, do you have any questions about the Healthy Minds Program app?

We will be reporting your feedback to the creators of this app so we want to learn about your use and thoughts about the app. We want to learn how to make the app feasible for people and we can only do that when we understand the realities of people who have never used it. You’re representative of the population we want to introduce this app to, so your input is very important.

In one word or phrase, describe your overall experience using the app in the last week.

I really want to know your general reactions to the app generally so, what would you like me to know about your use of the app?

How did you find the navigation of the app? How clear were the instructions?

Now we are going to do an anonymous poll.

*How many times in the last week have you used the app? Poll

What time of the day did you most use the app? - Poll

Do you mostly practice in a group? - Poll

End of poll session***

If you thought of using this app in the future, what would be your goal(s)?

With the existing features, would it help you achieve your goals? How?

Did you encounter any difficulties using the app? What were they?

What would you like the app creators to know about how to improve your use of the app? Or What would you like us to tell them to improve the app to meet your goals?

Which features of the app did you like?

Which features of the app did you like the least?

Which of the modules did you like best?

Which of the modules did you like least?

What is your opinion on the number of sessions in each module?

What is your opinion on the length of the sessions?

Before we end this meeting, is there anything else anyone would like to share about the Healthy Minds Program app?

Closing remarks

Thank you for joining us and sharing your thoughts and opinions about the Healthy Minds Program App. We really appreciate all the feedback you've shared. Your input will help generate a guideline for how clinicians can introduce the app to potential users. 

We will be initiating your payment right away so you should receive it in your email in 1-2 weeks. We are looking forward to speaking with you all again. Would you be able to meet again same time next week? (Have a list of alternatives ready) If you have any questions after this session, feel free to contact me at __________.

Fun activity before next week: Do one thing that you've always wanted to do for self-care.
